# Flow Enabled Target Capture Halbach‐based magnetic enrichment increases circulating tumor cell capture from blood in metastatic cancer patients

**DOI:** 10.1002/1878-0261.70267

**Published:** 2026-05-29

**Authors:** Michiel Stevens, Anouk Mentink, Tom Niessink, Frank Coumans, Leonie Mekenkamp, Ruchi Bansal, Jeroen Hiltermann

**Affiliations:** ^1^ Tzu Cancer Therapeutics Amsterdam The Netherlands; ^2^ Personalized Diagnosis and Therapeutics, Faculty of Science and Technology University of Twente Enschede The Netherlands; ^3^ Decisive Science Amsterdam The Netherlands; ^4^ Department of Internal Medicine Medisch Spectrum Twente Enschede The Netherlands; ^5^ Department of Medical Oncology University Hospital Groningen The Netherlands

**Keywords:** circulating tumor cells, EpCAM expression, liquid biopsy, magnetic separation

## Abstract

The clinical implementation of circulating tumor cells (CTCs) as a predictive tool for therapy efficacy in the growing field of precision oncology likely requires advanced CTC phenotyping. While their predictive value for disease progression and therapy response has extensively been shown, the low number of CTCs that are obtained from whole blood tubes limits their clinical application. The magnetic enrichment of CTCs using the FDA‐cleared CellSearch system is limited by the expression of the epithelial cell adhesion molecule (EpCAM) on CTC, and an increase in CTC yield may be obtained through enhanced capture of low EpCAM expressing cells. In this study, we compared the Flow Enabled Target Capture Halbach (FETCH) magnetic enrichment technology with the FDA‐cleared CellSearch system for CTC capture using paired blood samples from 34 patients with non‐small‐cell lung, breast, and prostate cancer. Results show a statistically significant median 1.5‐fold increase in CTCs captured with the FETCH system compared with CellSearch, accompanied by a median twofold reduction in non‐specifically captured leukocytes. This increase in CTC capture can aid the comprehensive analysis of CTCs in a larger number of patients, further supporting clinical implementation of CTC‐based diagnostics.

## Graphical abstract

Pair‐wise comparison of the CellSearch and FETCH enrichment technologies for circulating tumor cells (CTCs) from metastatic breast, prostate, and small cell lung cancer patients shows an increased capture of CTCs using FETCH enrichment.

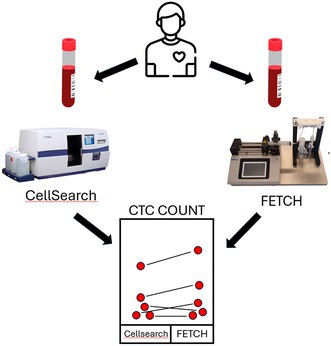

**Abbreviations**
ANOVA: analysis of variance
CD45: cluster of differentiation 45
CTCs: circulating tumor cells
EpCAM: epithelial cell adhesion molecule
FDA: U.S. food and drug administration
FETCH: flow enabled target capture halbach
NSCLC: non‐small‐cell‐lung‐cancer
PBS: phosphate buffered saline

## 1 Introduction

Circulating tumor cells (CTCs) are the main drivers of metastasis [1]. While enumeration of CTCs, predominantly using the FDA‐cleared CellSearch system, has extensively been validated as prognostic for disease progression and therapy response, prognostic stratification alone has not yet been sufficient for clinical implementation [2, 3, 4]. In the context of increasingly available targeted therapies [5], molecular analysis of the detected CTCs could add significant clinical value, at both diagnosis and progression of disease and in tackling resistant mechanisms. When sufficient CTCs are available, advanced CTC analysis technologies such as fluorescent detection of one or several antigen(s), bulk or single cell genomic or transcriptomic analysis, and functional or drug screening can be applied [6]. However, the clinical utilization of these methods is severely hampered by the typically low numbers of CTCs detected from a standard blood tube.

Immunomagnetic enrichment of CTC relies on the sufficient expression of an enrichment target on CTC, which should not be expressed on blood cells. The epithelial cell adhesion molecule (EpCAM) is widely expressed in epithelial tissue, but not in hematopoietic cells. However, some epithelial carcinoma may have low EpCAM expression due to poor differentiation or tissue type, and epithelial‐to‐mesenchymal‐transition [7, 8, 9, 10]. The FETCH immune–magnetophoretic technology was developed to increase the capture rate of EpCAM‐low expressing CTCs [11]. This technology uses a combination of microfluidics and strong magnetic fields to increase the magnetic attraction of CTCs. FETCH technology has previously been demonstrated using leukapheresis material [12], but has so far not been utilized for regular blood tube processing. Given that the expression of antigens is often a continuum rather than a complete absence or presence, we postulated that when using the same EpCAM targeting magnetic particles, the increased sensitivity of the FETCH system for cells with low numbers of bound particles could lead to an increase in the number of CTCs captured from regular blood tubes compared with CellSearch.

In this study, we therefore directly compared the capture of CTCs from blood tubes collected from patients with metastatic breast, prostate, and non‐small cell lung cancer (NSCLC) using the magnetic separation of FETCH and the FDA‐approved CellSearch system.

## 2 Methods

### 2.1 Patient samples

Samples from metastatic NSCLC patients were collected at the University Medical Center Groningen as part of the FETCHing NSCLC study (OLS053‐202211454), an OncoLiFeS cohort, approved by the medical ethical committee of the University Medical Center Groningen [13]. Metastatic breast and prostate cancer patient samples were collected at the Medisch Spectrum Twente as part of the FETCH‐CTC study (NL83876.000.23) approved by the medical ethical committee Oost‐Nederland. All samples were collected in accordance with the Declaration of Helsinki, and all patients gave written informed consent prior to inclusion.

Patients were included before the initiation of a new line of treatment. Samples were collected between April 2023 and September 2025. Blood samples were stored in CellSave vacutainers (Menarini, Bologna, Italy) and shipped by regular post to the University of Twente. Samples that arrived within the set time frame of 96 h were processed. Samples arriving after this timeframe were discarded.

### 2.2 CTC enrichment

From each patient, two samples were processed simultaneously, one with the CellSearch Autoprep system and one with the FETCH magnetic separation device.

#### 2.2.1 CellSearch

The sample processing using the CellSearch Autoprep system was performed according to the manufacturer's instructions. In brief, 7.5 mL of blood was supplemented with 6.5 mL of CellSearch dilution buffer (Menarini, Italy) and centrifuged for 10 min at 800× *g* without brake. The sample was then loaded into the CellSearch Autoprep system for immunomagnetic enrichment and immunofluorescent staining using the Circulating Tumor Cell Kit (Menarini, Italy).

#### 2.2.2 Fetch

For FETCH enrichment, the previously described FETCH enrichment device [14] was used. Here, 7.5 mL of blood was supplemented with 6.5 mL of CellBuffer, composed of PBS buffer (Merck, Darmstadt, Germany) supplemented with bovine serum albumin (Merck, Darmstadt, Germany), EDTA (Merck, Germany), casein (Merck, Germany), and mouse serum (Invitrogen, Carlsbad, USA). The sample was centrifuged for 10 min at 800 x *g* without brake. The resulting plasma layer was manually aspirated, leaving approximately 6 mL of sample.

Next, 3 mL of CellBuffer and 50 μL of CellSearch ferrofluid were added, and the sample was mixed by inversion. After a 3‐minute incubation, 50 μL of CellSearch capture enhancement was added, and the sample was incubated in a quadrupole magnet for eight times 3 min, mixed by inversion between incubations.

Separation was performed in the FETCH magnetic separation setup by pulling the sample through the separation flow chamber at 0.5 mL/min using a syringe pump. Next, the flow chamber was washed using 1 mL of CellBuffer at the same flow speed to remove residual uncaptured cells. Finally, the magnetic array was removed, and the collected sample was retrieved by flushing the chamber with 2 mL CellBuffer and air using a manually operated syringe. Enriched samples were collected in a 12 × 75 mm conical tube and magnetically separated for 5 min in an iMAG magnet (BD, Franklin Lakes, NJ, USA). The unbound fraction was then aspirated using a glass Pasteur pipette and the syringe pump set to 2 mL/min. After resuspension in 1 mL PBS BSA 1%, the sample was again magnetically washed and subsequently resuspended in 300 μL staining buffer.

#### 2.2.3 Staining

To allow comparison of capture sensitivity alone, the sample pairs of FETCH and CellSearch enriched samples were stained using reagents from the same CellSearch Kit lot numbers, eliminating the influence of reagents on our results. For this staining buffer consisting of 50 μL CellSearch nuclear stain, 50 μL CellSearch staining reagent, 50 μL CellSearch permeabilization reagent, 75 μL CellBuffer, and 75 μL PBS were used.

After 20 min of staining at RT, 700 μL CellBuffer was added and the sample was magnetically washed by separation for 10 min in an iMAG magnet, followed by aspiration of the unbound fraction and resuspension in 1 mL of CellBuffer. After another 5 min separation in an iMAG magnet and aspiration of the unbound fraction, the stained sample was resuspended in a mixture consisting of 150 μL of Cell Search Cell Fixative together with 200 μL PBS and placed into a CellSearch cartridge.

### 2.3 CTC scoring

As the intent of these studies was to compare the sensitivity in magnetic capture sensitivity, for both methods, the identification of CTCs was performed using the CTC‐scoring software of the CellSearch system according to the manufacturer's instructions. In short, the user selects CTC from an automatically generated gallery of candidate events based on predefined criteria, where a CTC is defined as having a nucleus, positive for cytokeratin, negative for CD45, and having a cell‐like morphology [15].

### 2.4 Total cell counting

While the aim is to capture as many CTCs as possible, an increase in sensitivity often coincides with a reduction in specificity. In the case of magnetic enrichment of CTCs, this increases the number of leukocytes that are co‐enriched. To evaluate the extent to which this occurs with both methods, the total number of nucleated events in the enriched samples was determined from the CellSearch archives using StarDist segmentation [16] in combination with a deep learning approach [17].

### 2.5 Statistics

Due to the non‐normal distribution of both CTC and total cell counts, paired Wilcoxon signed rank test was used to compare numbers of identified CTCs and total cells between both methods. To compare the CTC counts in the three different cancer types, the non‐parametric Kruskal–Wallis ANOVA was used. To be robust against the large variation in CTCs and total cells, linear regression was performed after log‐transformation of the data. Here, CTC + 1 counts were used to allow log‐transformation of zero CTC counts. For display purposes on logarithmic scales, we have used 0.1 to display zero CTC counts. All statistics were performed using Origin 2019B (OriginLab, Northampton, MA, USA).

## 3 Results

### 3.1 Patient inclusion and characteristics

**Fig. 1:**
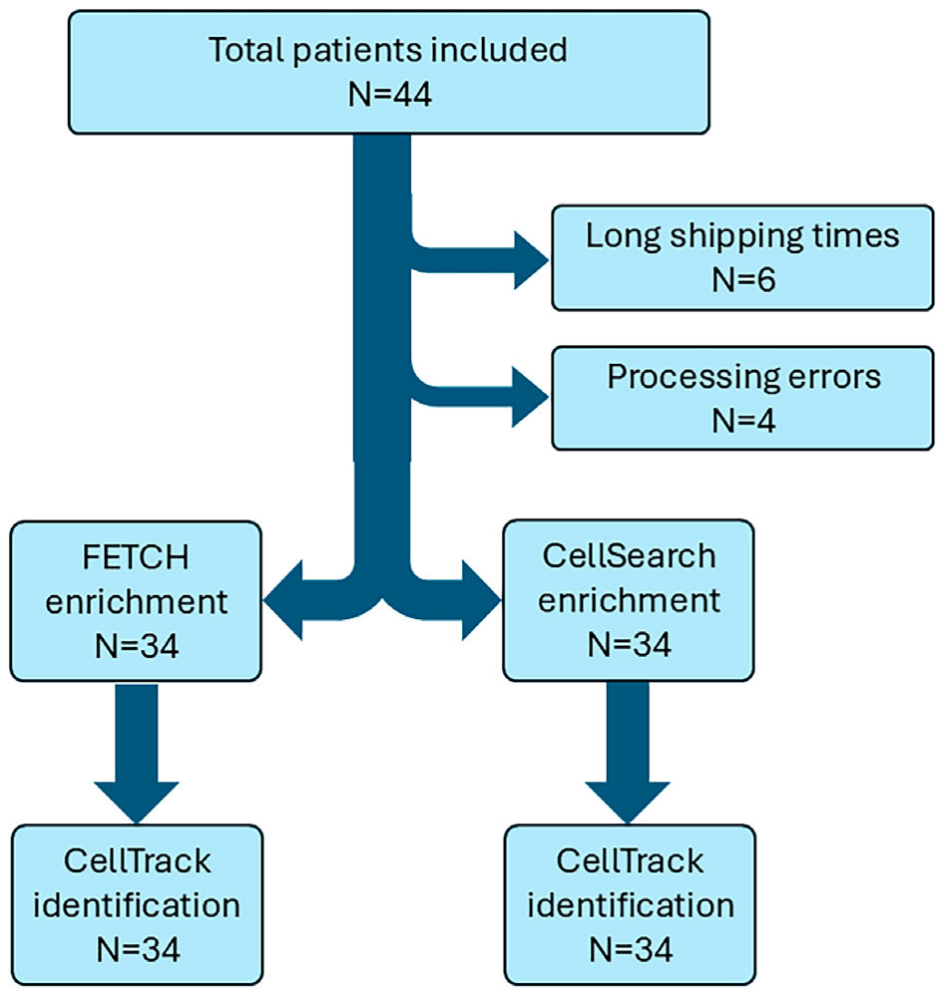
Study setup and inclusion. Schematic flow diagram of the study setup and inclusion.

Between April 2023 and September 2025, 27 non‐small cell lung cancer patients were included at the University Medical Center Groningen, and 11 breast‐ and 6 prostate cancer patients were included at the Medisch Spectrum Twente (Fig. 1). Samples from six patients were excluded as shipment by regular post took more than 3 days. Four samples were excluded due to either human or mechanical error during processing. For all included patients as well as those successfully processed and analyzed, general patient characteristics are shown in Table 1.

**Table 1:** Patient characteristics.

Characteristic		Total inclusion	In analysis
Age—mean (min‐max)		68 (36–83)	69 (36–83)
Sex—n (%)	Male	19 (43)	17 (50)
	Female	25 (57)	17 (50)
Tumor type—n (%)	Breast	11 (25)	9 (26)
	Prostate	6 (14)	4 (12)
	Lung (NSCLC)	27 (61)	21 (62)
Tumor stage—n (%)	IV	44 (100)	34 (100)
Previous therapies—n (%)	0	20 (45)	15 (44)
	1	11 (25)	11 (32)
	2	5 (11)	2 (6)
	3	6 (14)	5 (15)
	4	2 (5)	1 (3)

### 3.2 CTC recovery

**Fig. 2:**
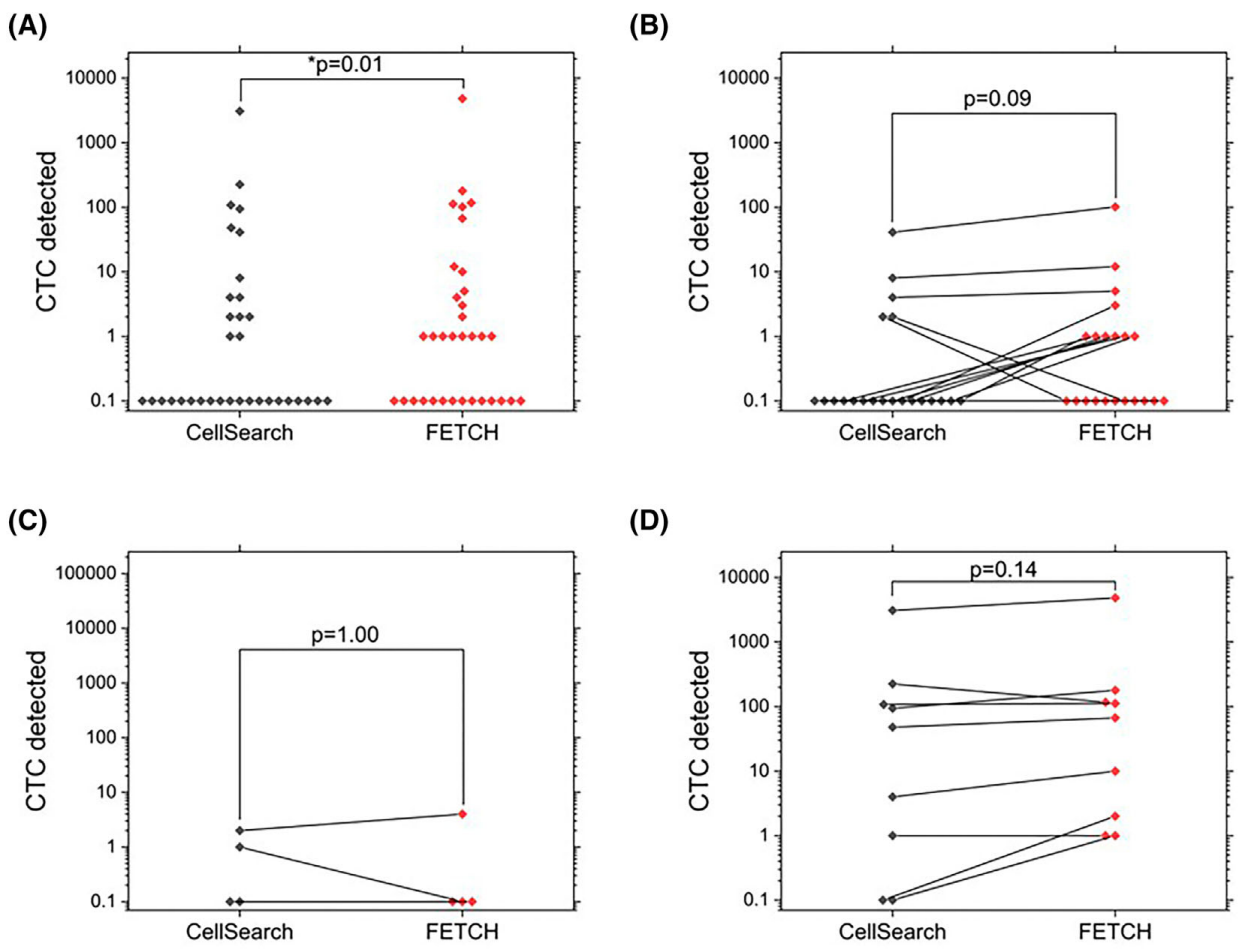
Circulating tumor cell (CTC) detection comparison between FETCH and CellSearch. Comparison of the number of CTCs detected in (A) all (*N* = 34) or only (B) NSCLC (*N* = 21), (C) prostate‐(*N* = 4), or (D) breast cancer (*N* = 9) patient tested sample pairs, showing an apparent increase in both breast and NSCLC patient samples. *Y*‐axis in log scale and sample pairs connected in panels B–D, * indicates a significant difference (*P* < 0.05) according to the Wilcoxon signed rank test.

The number of CTCs detected after FETCH enrichment was significantly higher than CellSearch (Wilcoxon signed rank test, *P* = 0.012), see Fig. 2A. An overview of all CTC counts is provided in Table S1. When separated per cancer type (Fig. 2B–D), the increase in CTC for FETCH enrichment over CellSearch is not significant in the individual types, but most apparent in NSCLC (*n* = 21) and breast cancer (*n* = 9) patient samples, containing the largest patient numbers. The difference in the number of CTCs detected per cancer type is not significant in these still small cohorts. As expected, the average number of CTCs detected in NSCLC was smaller than that in breast cancer (Kruskal–Wallis, *P* = 0.003). In this small dataset, the number of CTCs detected in prostate cancer (*n* = 4) was not significantly different from the other cancer types.

In pairs for which in one of the samples no CTCs were detected, it is impossible to determine a fold increase. In samples with low numbers, large sample sets are needed to correctly assess a fold change, as a change from one to two CTCs detected will often be a result of stochastic sampling. To obtain an indication of the level of CTC increase in this small data set, we examined the fold change in CTC for all sample pairs in which at least three CTCs were detected with both methods (*N* = 9). In these samples, the median increase obtained with FETCH enrichment compared with CellSearch enrichment was 1.5‐fold.

**Fig. 3:**
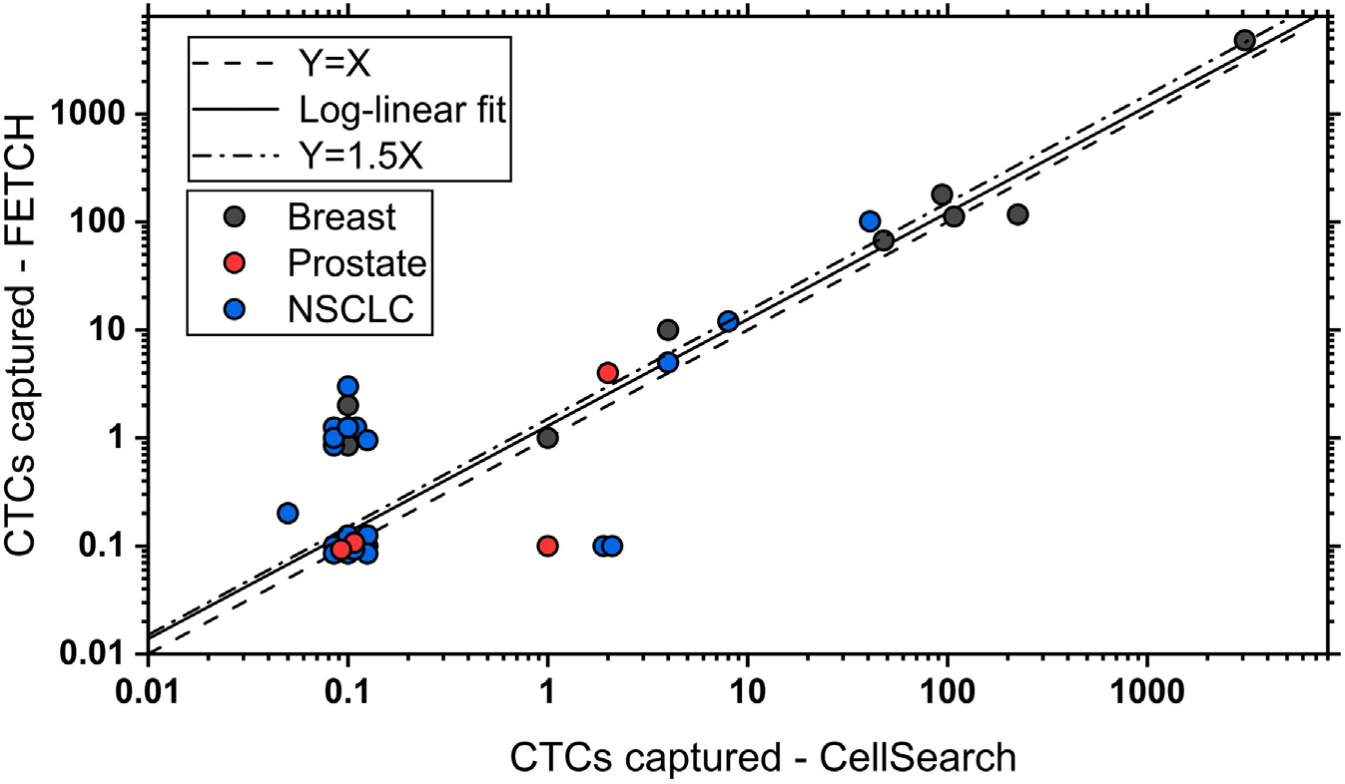
Comparison and log‐linear fit of circulating tumor cell (CTC) detected. Scatter plot showing the number of CTC obtained using CellSearch (x‐axis) and FETCH (y‐axis) enrichment from all 34 sample pairs. Linear fit on log‐transformed data shows a linear relation with log10(CTCFETCH+1) = 0.097 + 0.995 *log10(CTCCellSearch+1) with an R2 of 0.93. Samples from different types of cancer are indicated by color. Jitter was added to overlapping datapoints for visualization. The *Y* = 1.5X illustrates a 1.5‐fold increase on the used log‐scale representation.

The sensitivity for EpCAM‐high CTCs is expected to be similar between FETCH and CellSearch and only increased by the additional capture of EpCAM‐low CTC. Therefore, the number of CTCs captured by both methods is expected to be highly related. To assess this, we performed linear regression. Here, to be robust against the large difference in CTC numbers, we first log‐transformed CTC counts. Subsequent regression analysis resulted in a fit of log_10_(CTC_FETCH_ + 1) = 0.097 + 0.995 log_10_(CTC_CellSearch_ + 1) with an *R* ^2^ of 0.93 (Fig. 3), indicating indeed a high level of correlation between the number of CTCs found with both methods.

### 3.3 Co‐capture of healthy leukocytes

The co‐capture of leukocytes poses an important factor in the practical application of CTC enrichment. To see whether the increase in CTC capture is accompanied by an increase in non‐specific capture, the total number of cells captured for both methods was compared. An overview of the total number of captured cells for all samples is provided in Table S1. In addition to a significant increase in the number of captured CTCs, the number of false positives was significantly lower (Wilcoxon signed rank, *P* = 0.005) using the FETCH enrichment compared with CellSearch with a median 2.1‐fold decrease in detected leukocytes. As previously shown for CellSearch [16], linear regression of log‐transformed total cell number counts showed an increase in total cell numbers at longer time intervals between sample collection and processing for both FETCH (*P* < 0.01) and CellSearch (*P* < 0.01) processing.

**Fig. 4:**
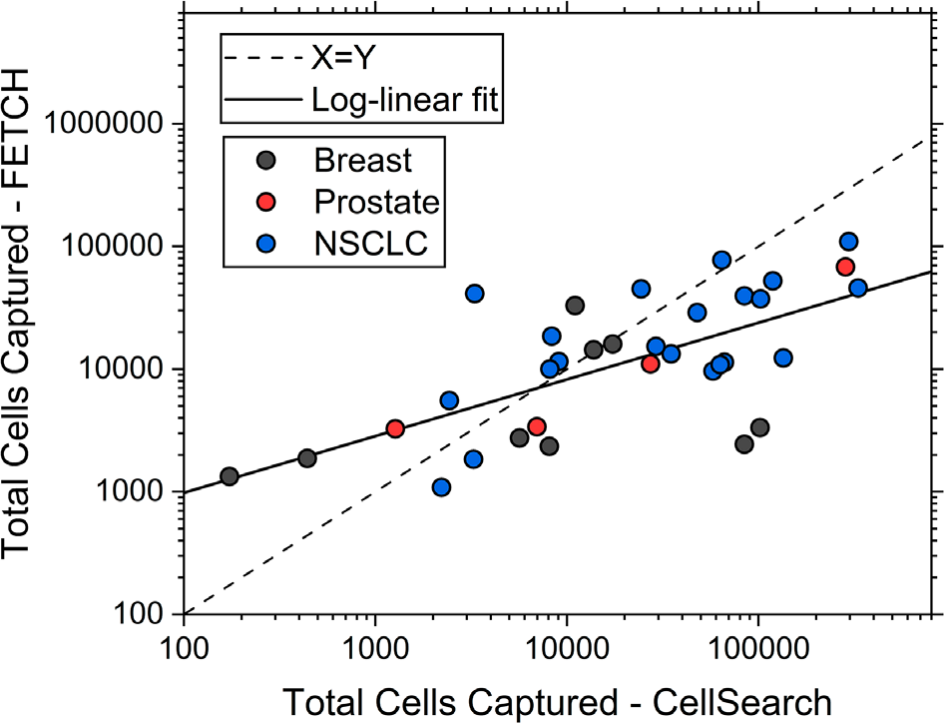
Comparison and log‐linear fit of the total number of captured cells. Scatter plot showing the total number of cells captured using CellSearch (x‐axis) and FETCH (y‐axis) enrichment from all 34 sample pairs. Linear fit on log‐transformed data shows a linear relation with log10(Total_CellsFETCH+1) = 2.06 + 0.47*log10(Total_CellsCellSearch+1) with an R2 of 0.45.

Moreover, although the number of CTCs captured was highly correlated between both methods, the total number of captured cells was only mildly correlated with a linear fit on log‐transformed data of log_10_(Total‐Cells_FETCH_ + 1) = 2.06 + 0.45 log_10_(Total‐Cells_CellSearch_ + 1) and an *R* ^2^ of 0.47, see Fig. 4.

## 4 Discussion

In this study, we demonstrated that the FETCH magnetic enrichment technology results in a 1.5‐fold increase in CTCs detected from blood tubes compared with CellSearch, along with a twofold decrease in non‐specific capture of healthy cells. This leads to a combined threefold increase in enrichment selectivity. The clinical significance of these additionally captured CTCs will still need to be proven, as we expect FETCH to recover lower‐EpCAM expressing cells than CellSearch [18].

While it was expected that the increase in sensitivity attained using the FETCH magnetic enrichment would also result in a larger background of non‐specifically captured cells, our results show a significant decrease in the number of co‐captured leukocytes when using the FETCH enrichment. This reduction is most likely due to the lower amount of ferrofluid used, increasing the specificity of the magnetic particle binding. In line with this reasoning, it is noted that in samples in which low numbers of cells are co‐captured with CellSearch (< 5000), the FETCH enrichment appears to increase non‐specific capture due to the increased sensitivity. In samples where more non‐specific binding occurs, the (threefold) lower concentration of magnetic particles used appears to limit the number of co‐captured white blood cells, likely via an increase in the labeling specificity.

In some sample pairs, more CTC are found in the sample processed with CellSearch. This is likely a result of random sampling according to the Poisson distribution, causing some samples from the same patient to have one or a few CTCs, while the other sample has no CTCs at all.

In samples pairs with > 2 CTCs detected in both samples (26%), the resulting median increase of 1.5‐fold will facilitate better and more reliable CTC‐based tumor characterization. However, given the still low number or even absence of CTCs detected in most patients (74%), an increase in the blood volume examined will likely be required for clinical application, possibly via the use of leukapheresis [12]. Here, the obtained reduction in the number of unwanted leukocytes captured will be most relevant for the practical feasibility of performing in‐depth analysis of the captured CTCs [19].

The clinical use of CTCs has been limited, primarily due to the low number of CTCs detected in most patients. For CTCs to be useful in the rapidly developing landscape of precision and even personalized therapy, in‐depth analysis will likely be needed, requiring a larger number of tumor cells. Especially in NSCLC, the large number of patients for which no CTCs are detected makes CTC‐guided expression analysis uninformative. In our cohort, CTC positivity using the CellSearch system was lower than previously reported in metastatic NSCLC [20] (24% (5/21) vs 33–36%, respectively) but increased to 48% (10/21) when FETCH enrichment was applied. Overall, the percentage of CTC‐positive samples in the complete cohort increased from 44% (15/34) to 59% (20/34) when using CellSearch and FETCH enrichment, respectively.

Due to the small sample size, we did not investigate the prognostic value of the CTCs captured using the FETCH enrichment in this study. However, the high correlation between the previously proven prognostic CellSearch counts and those obtained using the FETCH enrichment provides a positive indication of the prognostic significance of CTCs captured using the FETCH platform.

## 5 Conclusion

The FETCH enrichment technology demonstrates a 1.5‐fold increase in CTCs captured from blood samples of metastatic cancer patients compared with CellSearch. Additionally, the number of co‐captured white blood cells is reduced by twofold, increasing the resulting sample purity. This increase in both the number and purity of detected CTCs can contribute to the capture of sufficient CTCs for in‐depth tumor characterization and clinical use. However, larger volume screening, through multiple blood tubes or leukapheresis, will likely still be required for most patients.

## Conflicts of interest

MS and TN are current employees of Tzu Cancer Therapeutics, which is further developing the FETCH technology for circulating tumor cells analysis. FC is the founder and owner of Decisive Science BV. Other authors declare no conflict of interest.

## Author contributions

MS contributed to the conceptualization. FC and MS contributed to the formal analysis. RB, LM, and JH contributed to the resources. AM and TN contributed to the investigation. FC and RB contributed to the software. MS contributed to the writing—original draft. All authors contributed to the writing—review and editing.

## Supporting information


**Table S1.** Overview of all CTC and total cell counts for the analyzed sample pairs.

## Data Availability

All data including original TIFF images used for CTC and total cell counts are available from the corresponding author upon reasonable request.
