# Supplementary material for: Flow Enabled Target Capture Halbach‐based magnetic enrichment increases circulating tumor cell capture from blood in metastatic cancer patients
Source: Mol Oncol. 2026 May 29:10.1002/1878-0261.70267. Online ahead of print. doi: 10.1002/1878-0261.70267 (PMC13398564; doi:10.1002/1878-0261.70267)
Supplement: Supplementary file 1 — Table S1. Overview of all CTC and total cell counts for the analyzed sample pairs. [file MOL2-9999-0-s001.docx]

Supplementary materials

Flow Enabled Target Capture Halbach based magnetic enrichment increases circulating tumor cell capture from blood in metastatic cancer patients

Michiel Stevens^1,2 *^, Anouk Mentink^2^, Tom Niessink^1,2^, Frank Coumans^3^, Leonie Mekenkamp^4^, Ruchi Bansal^2^ and Jeroen Hiltermann^5^

**Table S1. Overview of all CTC and total cell counts for the analyzed sample pairs.**

| Patient # | Cancer type | # previous therapies | CTC | | Total Cells | |
| --- | --- | --- | --- | --- | --- | --- |
|  |  |  | CellSearch | FETCH | CellSearch | FETCH |
| 1 | Breast | 1 | 108 | 112 | 5664 | 2744 |
| 2 | Breast | 1 | 225 | 117 | 84509 | 2433 |
| 3 | Breast | 0 | 0 | 1 | 8094 | 2345 |
| 4 | Breast | 4 | 94 | 179 | 17332 | 16009 |
| 5 | Breast | 3 | 48 | 67 | 13794 | 14335 |
| 6 | Breast | 2 | 4 | 10 | 101960 | 3332 |
| 7 | Breast | 0 | 1 | 1 | 441 | 1867 |
| 8 | Breast | 3 | 3077 | 4813 | 11036 | 32907 |
| 9 | Breast | 0 | 0 | 2 | 173 | 1329 |
| 10 | NSCLC | 0 | 0 | 1 | 3306 | 41174 |
| 11 | NSCLC | 0 | 0 | 0 | 24420 | 44957 |
| 12 | NSCLC | 1 | 0 | 3 | 57955 | 9614 |
| 13 | NSCLC | 0 | 0 | 0 | 35036 | 13268 |
| 14 | NSCLC | 1 | 41 | 101 | 47864 | 28856 |
| 15 | NSCLC | 1 | 0 | 1 | 64512 | 77165 |
| 16 | NSCLC | 1 | 2 | 0 | 8328 | 18529 |
| 17 | NSCLC | 1 | 0 | 1 | 66506 | 11354 |
| 18 | NSCLC | 1 | 8 | 12 | 84515 | 39548 |
| 19 | NSCLC | 0 | 0 | 0 | 9084 | 11510 |
| 20 | NSCLC | 0 | 0 | 0 | 8150 | 9981 |
| 21 | NSCLC | 0 | 0 | 0 | 118810 | 52326 |
| 22 | NSCLC | 3 | 4 | 5 | 102582 | 37259 |
| 23 | NSCLC | 1 | 0 | 1 | 29173 | 15272 |
| 24 | NSCLC | 0 | 0 | 0 | 3259 | 1840 |
| 25 | NSCLC | 3 | 0 | 0 | 2433 | 5527 |
| 26 | NSCLC | 2 | 0 | 1 | 134926 | 12296 |
| 27 | NSCLC | 0 | 0 | 0 | 296252 | 109358 |
| 28 | NSCLC | 0 | 2 | 0 | 331669 | 45718 |
| 29 | NSCLC | 0 | 0 | 1 | 63153 | 10865 |
| 30 | NSCLC | 0 | 0 | 0 | 2216 | 1084 |
| 31 | Prostate | 1 | 1 | 0 | 27308 | 11016 |
| 32 | Prostate | 0 | 2 | 4 | 6970 | 3384 |
| 33 | Prostate | 0 | 2 | 0 | 284979 | 67981 |
| 34 | Prostate | 3 | 0 | 0 | 1271 | 3257 |
